# Fluoropolymers across Their Life Cycle: Why “Emissions Reduction” Is Not “Safe”

**DOI:** 10.1021/acs.est.6c12936

**Published:** 2026-10-14

**Authors:** Romain Figuière, Ian T. Cousins, Rainer Lohmann, Anna J. Miller, Amanda Rensmo, Martin Scheringer, Xenia Trier, Zhanyun Wang, Gabriel Sigmund

**Affiliations:** † Department of Environmental Science, 7675 Stockholm University, Stockholm SE-10691, Sweden; ‡ Graduate School of Oceanography, 54083 University of Rhode Island, Narragansett, Rhode Island 02882-1197, United States; § Institute for Biogeochemistry and Pollutant Dynamics, ETH Zurich, 8092 Zurich, Switzerland; ∥ University of Copenhagen, Department of Plant and Environmental Sciences, Section for Environmental Chemistry and Physics, Thorvaldsensvej 40, Frederiksberg C DK-1871, Denmark; ⊥ Department of Green Technology and Danish Institute for Advanced Study (DIAS), University of Southern Denmark, Odense 5230, Denmark; # Environmental Technology, 4508 Wageningen University & Research, Wageningen 6700 AA, The Netherlands

**Keywords:** PFAS, incineration, fluoropolymer manufacturing, residual emissions

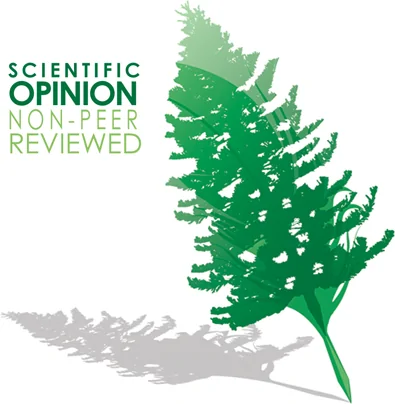

In the context of the proposal for a universal restriction on per- and polyfluoroalkyl substances (PFASs) in the European Union, recent industry reports argue that all uses of fluoropolymers (FPs) should be excluded from such restrictions.^1,2^ They claim that FPs are nontoxic, nonbioaccumulative, nonmobile, and that modern technologies can effectively control emissions of PFAS building blocks and processing aids at the manufacturing stage, as well as PFAS breakdown products at the end-of-life stages. By citing destruction and removal efficiencies (DREs) as high as 99.99%, these reports conclude that FPs pose a “negligible” risk across their life cycle.

According to an extensive body of independent evidence,^3–5^ these claims rely on flawed assumptions.

1. Optimized, case-specific technologies capable of achieving 99.99% DRE are not uniformly deployed across PFAS manufacturing facilities (i.e., producing FPs), nor across downstream processing facilities that process, compound, or fabricate fluoropolymers into finished products, or across recycling, incineration, and other end-of-life operations.
2. Current industry commitments focus narrowly on emissions of PFAS processing aids rather than the wider suite of feedstock chemicals, reaction intermediates, and transformation products that can be released during FP production, processing, and end-of-life disposal.
3. Even very small *relative* residual emissions (in percentages) are not negligible in terms of absolute mass of emitted PFAS, given the large scale of FP production and use, which further adds to PFAS accumulation in the environment.
4. Only a small fraction of FP can be separated and recycled, and only negligible amounts of waste are routed to facilities capable of achieving high DREs.

Taken collectively, these points make clear that high theoretical DRE values do not equate to “negligible” emissions across the fluoropolymer lifecycle. Consequently, there are substantial, scattered emissions of PFASs and other fluorinated substances, including feedstock monomers, processing aids, transformation products, products of incomplete destruction (PIDs)^6^ and other hazardous chemicals that occur through the FP life-cycle.

Since the beginning of the discussions on the need for a universal PFAS restriction aiming to effectively manage risks from PFAS pollution, manufacturers have claimed that FP production can meet average emission targets of 0.009% PFAS residues emitted to air and 0.001% to water, with a commitment to further reduce these values across Europe to 0.003% and 0.0006%, respectively, by 2030.^1,7^ A recent industry report states that “[t]­he Fluoropolymer Products Group of Plastics Europe has committed to aggressively manage emissions to water and air from the manufacture of fluoropolymers, has met their current commitments and is actively engaging with stakeholders”.^1^ To our knowledge, it is not clear whether these commitments have been fulfilled for each manufacturing site or only on average across sites in Europe, which could mean that some sites are still above these limits.

Although such reductions would represent substantial improvements compared to historical emissions, these figures may not reflect the regulatory and technical reality across all manufacturing sites. Some facilities already operate under strict, legally binding emission limits set at the national level (e.g., Chemours Dordrecht), whereas others face far more lenient requirements, resulting in substantial regional variation in actual PFAS releases.^3,8^

It is also unclear if the treatment controls are limited to PFAS building blocks and processing aids, which have been the primary focus of attention. Independent studies^3,5^ show that a broad range of PFASs and other fluorinated substances, beyond PFAS building blocks and processing aids, have been released across manufacturing processes, including unreacted raw materials and intermediates, oligomers, byproducts from monomer synthesis and polymerization, and other reagents, such as chain transfer agents or solvents.^3^ Based on field measurements, a study demonstrated that the emissions factors at the Dordrecht facility in The Netherlands are substantially lower than the global average factors, highlighting considerable variability in emissions across production sites and regions.^9^ These PFASs and other fluorinated substances differ substantially in volatility, mobility, and environmental fate, and many are not routinely monitored or regulated. As discussed elsewhere, focusing emission controls solely on PFAS building blocks and processing aids therefore overlooks a substantial portion of the environmental burden associated with FP production.^4^

Without comprehensive monitoring and binding limits covering all emissions during manufacturing, reported best-case DREs may be insufficient to reflect actual releases. Moreover, voluntary commitments may be unevenly implemented, and without binding emission standards applied uniformly across all facilities, the most ambitious DRE figures may not be representative of actual performance.

Even if the most ambitious emission reductions were universally achieved, the scale of current FP production means that residual emissions would still be substantial. With current FP production volumes estimated at 40–95 kt/y in the EU,^10^ even a residual emission fraction of 0.003% corresponds to annual releases of several thousand kilograms of PFASs. Treatment costs for PFASs in soil and water typically range between 1 and 10 Million €/kg PFAS,^11^ meaning that even these “low” emission scenarios would impose a substantial societal financial burden in addition to human and ecosystem health concerns. Such releases cannot reasonably be described as negligible, safe, or manageable.

Furthermore, although the evidence base remains limited, existing studies show that the downstream processing of FPs may also release PFASs.^4^ In particular, FP dispersions may contain elevated levels of nonpolymeric PFASs. They are processed into a wide range of products, typically involving a final high-temperature sintering step that can decompose some nonpolymeric PFASs while potentially generating or introducing others.^4,6^ Consequently, nonpolymeric PFASs can be released throughout processing via waste streams before sintering, as well as through air emissions during subsequent heating by downstream users. A recent experimental study has shown that thermal processing of FP dispersions can generate and release a variety of fluorinated compounds, including tetrafluoroethylene and other PFASs, under industry-relevant conditions.^12^ Less is known about potential releases during the use stage, but PTFE microplastics have been identified in many regions of the world,^4^ and their sources remain to be fully elucidated.

At the end-of-life stage, industry-funded studies claim that FP incineration can achieve DREs of 99.99% under optimized conditions.^13^ However, while this holds true for incinerators operating at temperatures above 850 °C with a sufficiently long incineration time, particularly those exceeding 1 000 °C and including fume gas treatment, PFAS products of incomplete combustion are commonly formed at lower temperatures and PFAS transformation pathways during incineration remain poorly understood.^14,15^ As such, hazardous and industrial waste incinerators can achieve high DREs, but it remains unclear how many regular municipal waste facilities can reach such a high performance. This distinction matters, as approximately one-third of FPs in circulation on the EU market are used for consumer applications, primarily in textiles and food contact materials.^10^ These products enter mixed household waste streams, which are routed toward municipal waste facilities rather than hazardous waste facilities. Ensuring that all FP-containing waste is treated in high-performance incineration facilities would require a high level of waste tracking and sorting that is not achievable at the European scale.

Even with the most optimistic assumption that 100% of the FP waste is treated in high-performance incinerators capable of achieving a DRE of 99.99%, the resulting PFAS emissions at the European scale are still not negligible.^10^ For instance, approximately 23.5 kt of FP waste was collected in 2020 in the EU, primarily through mixed waste streams or partially source-separated fractions, out of which 84% (approximately 19 740 tonnes) were incinerated.^16^ The optimistic DRE of 99.99% during the incineration of this FP waste would still result in an emission of 1.97 tonnes of PFASs at the EU level in a single year, from FP incineration alone. Due to the extreme persistence of PFASs in the environment, every emitted gram of PFAS matters. Thus, a 0.01% residual emission fraction from dealing with FPs at the end of life amounting to tonnes of PFASs cannot be considered “safe” nor “negligible”. Moreover, in many parts of the world, substantial quantities of waste are still landfilled, openly dumped, or burned at lower temperatures.

Consequently, the claim that FPs warrant a blanket derogation from any PFAS restriction or group-based regulation because their life-cycle emissions can be “safely controlled” is not scientifically supported by evidence. Apart from the limited coverage of PFASs and life-cycle stages, the DRE figures used to support this argument reflect optimized performance under controlled conditions that are unlikely to be representative of actual manufacturing or end-of-life management practices. The assumption that waste routing can be controlled sufficiently to ensure high-performance incineration for FP-containing waste is not realistic at scale. Even under the optimistic assumption of DREs of 99.99%, the absolute volume of PFASs emitted annually at the European scale from FP production and incineration is estimated to amount to tonnes per year, which is a substantial burden to human and ecosystem health, as well as public finances, which ultimately will pay the price for remediation and treatment in many cases.

It is also worth noting that while some uses of FPs remain challenging to substitute (e.g., in the medical, energy, military, semiconductor and aerospace sectors), a substantial proportion of FP uses already have commercially available nonfluorinated alternatives.^17,18^ Research has also shown that substitution of FP is possible for certain components even within a “challenging” sector.^19^ A blanket derogation for all FPs which treats these uses identically is thus not justified nor consistent with how the REACH restriction process handles derogations.^20^ A more appropriate regulatory response would be to restrict all PFASs, including FPs, with time-limited derogations for specific uses where non-PFAS alternatives are not currently available, thereby providing a defined transition period for safer solutions to be developed and marketed. The ultimate objective of restricting production and use of PFASs should not be to minimize emissions. It should be to eliminate them.
